# Total knee arthroplasty and medial patellofemoral ligament reconstruction for knee osteoarthritis with habitual patellar dislocation: a case report

**DOI:** 10.3389/fsurg.2025.1629641

**Published:** 2025-10-01

**Authors:** Hanyu Zhou, Hongguang Zhu, Duanbo Lv, Qiaolin Liu, Yinghao Zheng, Facai Lin, Jian Li, Zhenlong Xing

**Affiliations:** 1 Affiliated Guangdong Hospital of Integrated Traditional Chinese and Western Medicine of Guangzhou University of Chinese Medicine, Foshan, China; 2 Department of Joint Surgery, Guangdong Provincial Hospital of Integrated Chinese and Western Medicine, Foshan, Guangdong, China; 3 School of Acupuncture and Tuina, School of Health and Rehabilitation, Nanjing University of Chinese Medicine, Nanjing, China

**Keywords:** total knee arthroplasty, medial patellofemoral ligament reconstruction, knee osteoarthritis, habitual patellar dislocation, case report

## Abstract

**Background:**

Knee osteoarthritis and habitual patellar dislocation are clinically common osteoarticular diseases. However, existing literature lacks reports on their co-occurrence and treatment approaches. Thus, we propose a surgical approach that integrates total knee arthroplasty with medial patellofemoral ligament reconstruction, addressing knee osteoarthritis alongside habitual patellar dislocation.

**Case presentation:**

A 78-year-old female developed habitual lateral patellar dislocation of the left knee after an accidental fall over 20 years ago. One year prior, she experienced weight-bearing pain in the left knee. Physical examination, full-length lower limb radiography, and computed tomography confirmed a diagnosis of left knee osteoarthritis with habitual patellar dislocation. The patient subsequently underwent total knee arthroplasty combined with medial patellofemoral ligament reconstruction.

**Conclusion:**

This case demonstrates notable innovation among similar reports. It may represents an effective treatment, enabling the patient to walk without impediment, with a stable and pain-free knee joint and no significant patellar displacement during flexion. Further case studies are still required to validate the generalizability of this surgical technique.

## Introduction

Total knee arthroplasty (TKA) is widely used clinically for treating end-stage knee osteoarthritis (KOA). Patellar dislocation or instability can also occur alone or following TKA, and literature reports indicate that medial patellofemoral ligament reconstruction (MPFLr) is effective in addressing post-TKA patellar dislocation or malalignment (1, 2). However, clinical cases of KOA combined with habitual patellar dislocation (HPD) are extremely rare. Previous studies have reported a limited number of cases where TKA was combined with lateral patellar retinaculum release and medial retinaculum plication to treat KOA with fixed patellar dislocation (3–5), but research on cases involving HPD remains scarce. Therefore, based on previous research, we innovatively explored whether the simultaneous application of TKA and MPFLr could yield favorable clinical outcomes in treating KOA combined with HPD.

### Patient background

The patient is a 78-year-old female who experienced an accidental fall while walking 20 years ago but did not receive systematic treatment. Subsequently, the patella dislocates laterally when the left knee is flexed and reduces upon knee extension, accompanied by significant pain that improves after rest, though symptoms recur repeatedly. A year ago, she began experiencing worsening pain in her left knee joint, which became particularly noticeable while walking and made it difficult to go up and down stairs. The patient reflected that this pain severely impacted her life. The patient had a history of left proximal femoral nail antirotation(PFNA) for a left femoral shaft fracture over 20 years ago. She denied any history of other medical or surgical conditions, any history of infectious diseases, and any allergies to food or medication.

**Figure 1:**
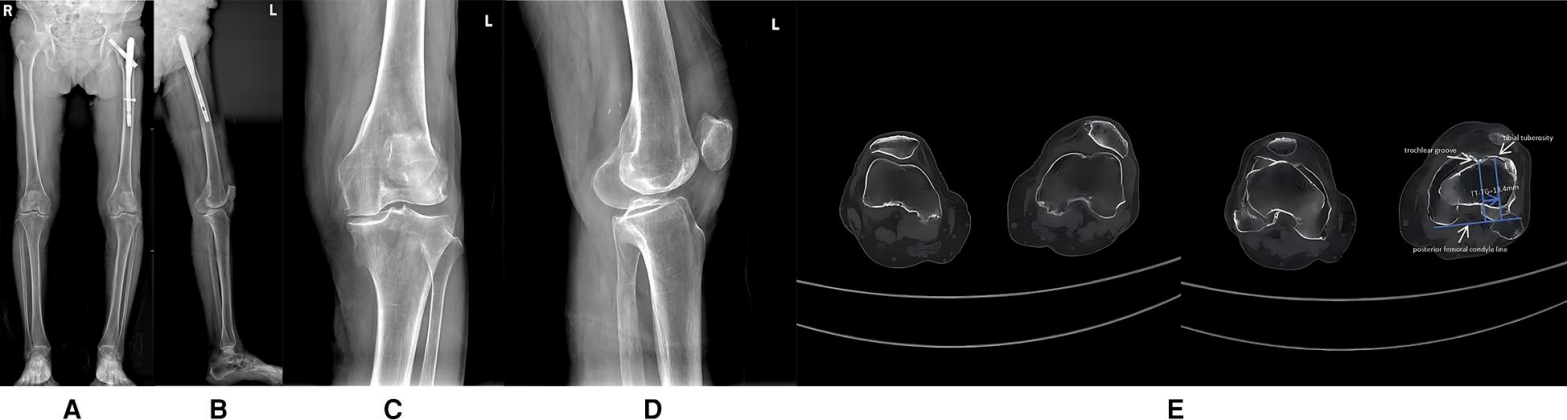
**(A)** Full-length frontal radiograph of the lower limb; **(B)** Full-length lateral radiograph of the lower limb; **(C)** Frontal radiograph of left knee joint; **(D)** Lateral radiograph of left knee joint; **(E)** CT axial images of left knee joint. [The tibial tuberosity–trochlear groove (TT-TG) distance is 13.4 mm].

Clinical examination revealed a mild varus deformity of the left lower limb, with approximately 10° of varus alignment. Mild tenderness was noted over the medial patella. The active range of motion of the left knee joint was 0–110°, while passive range reached 0–120°. Patellar dislocation occurred when the knee is flexed at 40° (refer to Supplementary Video S1), with tolerable pain and a visual analogue scale (VAS) score of 5 (6), the knee society score (KSS) clinical score was 57, functional score was 15, and the Oxford Knee Score (OKS) was 41. When the joint is extended, the patella can be reduced; however, forced reduction impedes further knee flexion. The knee remained stable under varus and valgus stress, with negative anterior and posterior drawer tests at 90° of flexion. Full-length frontal radiograph of the lower limb and frontal radiograph of left knee joint demonstrated degenerative changes in both knees, including medial compartment narrowing and multiple osteophyte formation. Possible osteochondromas were noted at the medial upper tibial margins bilaterally, along with bilateral knee varus and patellar dislocation (refer to Figures 1A–D). Computed tomography (CT) further validated the aforementioned diagnosis (refer to Figure 1E).

Based on physical and imaging findings, the patient's current primary diagnoses are left KOA, left knee varus deformity, and left HPD. The patient desires to alleviate left knee pain, meet general functional requirements, and improve joint function and quality of life. Therefore, we performed simultaneous left TKA combined with MPFLr on the patient.

### Surgical procedure

#### Total knee arthroplasty

The patient was placed in the supine position after intraspinal anesthesia, with a tourniquet applied to the left limb, followed by routine disinfection and draping. Recheck the patient's passive knee flexion status (refer to Supplementary Video S2). The Insall anterior midline knee incision was employed, sequentially incising the skin and subcutaneous tissue. The medial patellofemoral ligament was absent and was replaced by adipose tissue. A deep medial parapatellar approach was used, leaving approximately 0.5 cm of fascia on the patellar side for suturing, and the joint capsule was entered via an incision through the vastus medialis muscle. After everting the patella and flexing the knee joint, significant cartilage degeneration can be observed, with severe wear on the medial and lateral femoral condyles accompanied by osteophyte formation. Additionally, femoral trochlear dysplasia and groove-like wear on the patellar articular surface are evident. Hyperplastic synovium, residual menisci, and the anterior and posterior cruciate ligaments were excised, along with osteophytes and loose bodies. Osteotomy was performed using measured resection combined with the gap-balancing technique. First, distal femoral osteotomy was performed with 6° of valgus and a distal resection thickness of 9 mm. Tibial osteotomy was performed using extramedullary guidance, adjusting the tibial rotational axis according to the Akagi line. The proximal end of the extramedullary guide was positioned two fingerbreadths from the tibial anterior spine, and the distal end was three fingerbreadths from the tibial anterior spine to adjust the posterior tibial plateau slope angle. The resection thickness of the lateral tibial plateau was 9 mm, with the osteotomy performed along the vertical long axis of the tibia. Upon completion, the gap was found to be satisfactory upon extension. Measure the anteroposterior diameter of the femur, perform a four-in-one osteotomy using osteotomy plate No. 3, followed by intercondylar notch osteotomy. After completing the osteotomy, measure the tibia using mold No. 2, and shape the proximal tibia with the corresponding tibial keel wing. Following appropriate soft tissue release, insert femoral mold No. 3, tibial mold No. 2, and a 9 mm spacer mold. Assess the left lower limb alignment, knee joint stability, and flexion-extension gap balance, which are satisfactory. Pulsed lavage of the knee joint was performed, followed by mixing the bone cement and sequentially implanting the No. 3 femoral prosthesis (Smith & Nephew, London, United Kingdom) and the No. 2 tibial prosthesis (Smith & Nephew, London, United Kingdom). Excess bone cement was cleared, the patella was trimmed and denervated, and after the bone cement solidified, a 9 mm polyethylene liner was implanted. Release the lateral patellar retinaculum and vastus lateralis tendon. The medial joint capsule is overlapped and clamped with a towel clamp forceps at the medial side of the patella. The patella remains dislocated upon knee flexion during the operation. The no-thumb test is negative during intraoperative assessment, indicating insufficient medial restraint. Consequently, a MPFLr is performed.

#### Medial patellofemoral ligament reconstruction

Appropriately extend the distal incision, perform blunt dissection on the pes anserinus. Avoid damaging the infrapatellar branch of the saphenous nerve. Isolate the semitendinosus, trim any excess branches with fine scissors, and harvest the tendon using a tendon stripper. Finally, suture both the proximal and distal ends of the tendon with high-strength sutures. The autologous tendon graft measures approximately 6 cm in length, with the suture threads left for later use.

**Figure 2:**
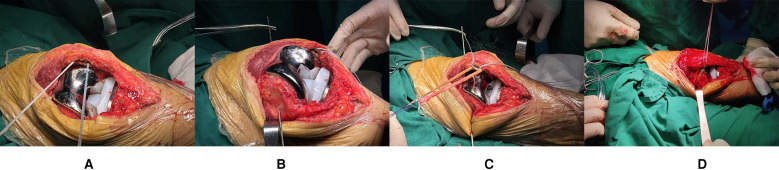
**(A)** A fresh bone trough was created at the proximal-middle 1/3 of the superomedial patellar border, and two PEEK suture anchors were implanted; **(B)** The femoral-side positioning point was selected as the midpoint between the medial femoral epicondyle and the adductor tubercle during the procedure; **(C)** The tendon was sutured and ligated into the patellar-side bone trough; **(D)** Intraoperative assessment showed favorable patellar tracking, and the negative “no thumb test” was confirmed.

**Figure 3:**
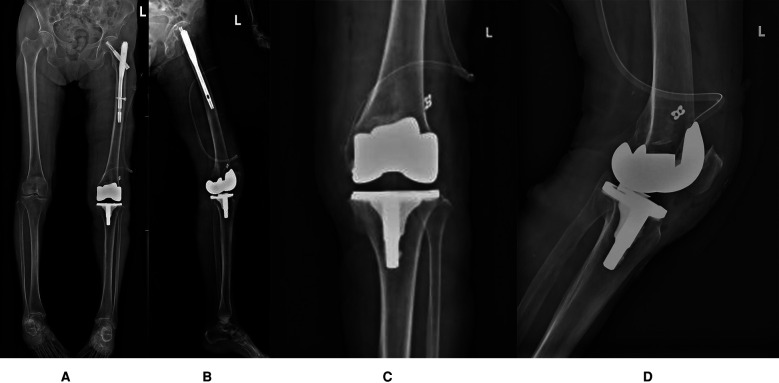
**(A)** Full-length frontal radiograph of the lower limb (Postoperative radiograph); **(B)** Full-length lateral radiograph of the lower limb (Postoperative radiograph); **(C)** Frontal radiograph of left knee joint (Postoperative radiograph); **(D)** Lateral radiograph of left knee joint (Postoperative radiograph).

At the proximal half of the medial superior border of the patella, freshen a bone trough and insert two 5.5 mm PEEK suture anchors (Rejoin, Hangzhou, China) at the mid-half and upper third of the patella (refer to Figure 2A). The positioning on the femoral side is performed using the palpation method, selecting the midpoint between the medial femoral epicondyle and the adductor tubercle as the target point, and positioning by guide pin (refer to Figure 2B). The tendon is then sutured and secured into the patellar bone trough (refer to Figure 2C). After positioning, drill a 6 mm cannulated reamer to the lateral cortex, pass the sutures through, and fix them on the lateral cortex using a four-leaf clover titanium plate (Rejoin, Hangzhou, China). Intraoperative testing indicates a negative “no thumb” sign, patellar tracking is satisfactory (refer to Figure 2D), the joint is stable, full knee extension reaches 0°, and flexion reaches 120°. The medial patellar joint capsule undergoes overlapping suturing, and the incision is closed in layers. Postoperative x-rays confirm proper implant positioning (refer to Figures 3A–D).

#### Postoperative rehabilitation

**Figure 4:**
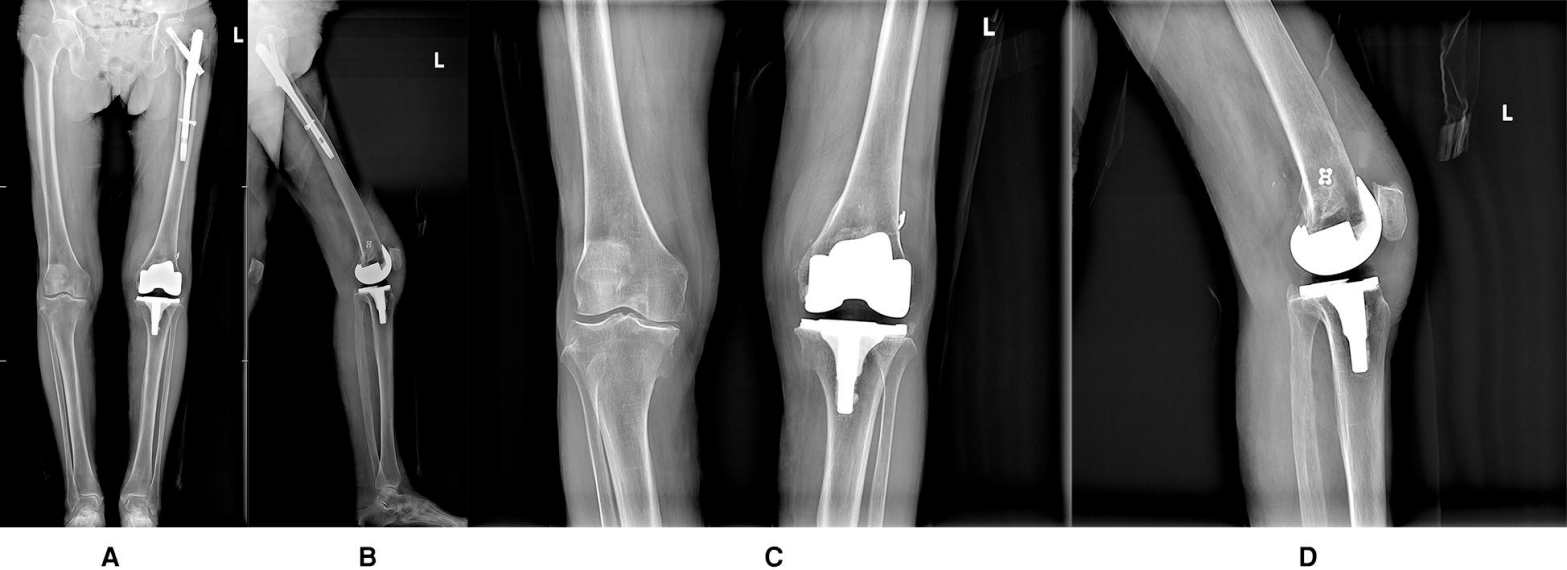
**(A)** Full-length frontal radiograph of the lower limb (The 7-month postoperative follow-up radiograph); **(B)** Full-length lateral radiograph of the lower limb (The 7-month postoperative follow-up radiograph); **(C)** Frontal radiograph of left knee joint (The 7-month postoperative follow-up radiograph); **(D)** Lateral radiograph of left knee joint (The 7-month postoperative follow-up radiograph).

The affected limb was placed in a hinged knee brace locked in extension. Ankle pumps and quadriceps strengthening exercises were initiated on postoperative day 2. Knee flexion exercises commenced 1 week postoperatively, with ambulation assisted by a walker and maintaining straight leg raises. The surgical incision healed by first intention, and sutures were removed two weeks postoperatively.At the one-month follow-up, the patient continues with flexion-extension exercises and quadriceps strengthening exercises. The patient reflected subjective satisfaction, ambulating with walker assistance and experiencing mild pain (with a VAS score of 3) after walking, which resolved with rest. The KSS clinical score was 85, functional score was 60, OKS score was 28. The left knee joint was stable with active flexion-extension range of 0–90°. By the 2-month follow-up, the patient expressed high subjective satisfaction, demonstrating normal gait without significant pain (with a VAS score of 1). The KSS clinical score was 90, functional score was 80, OKS score was 15. The left knee remained stable with active flexion-extension range of 0–100° (refer to Supplementary Video S3). The most recent follow-up, conducted seven months postoperatively, revealed no complications, including prosthetic loosening, periprosthetic infection, or lower extremity thrombosis. The patient expressed high satisfaction with the surgical outcome.The patient demonstrated unrestricted walking ability, reported no significant pain in the affected limb (with a VAS score of 0), and exhibited good functional performance during left knee flexion-extension without notable patellar displacement. The KSS clinical score was 93, functional score was 90, OKS score was 12. A follow-up radiograph confirmed that the prosthesis's position is optimal (Figures 4A–D).

## Discussion

Osteoarthritis is a chronic degenerative joint disease characterized by pain, swelling, stiffness, and dysfunction (7). TKA serves as the definitive treatment for end-stage KOA, effectively alleviating pain, reconstructing joint structure, and improving mobility (8). KOA often coexists with patellofemoral joint pathologies, including lateral patellar compression, patellofemoral joint wear, and even patellofemoral subluxation (9). For KOA complicated by patellofemoral disorders, satisfactory clinical outcomes can typically be achieved through increased measures such as internal femoral rotation osteotomy, external rotation placement of the tibial component, and medial placement of the patellar component, supplemented when necessary by retinaculum patellae laterale release or medial retinaculum plication (10–12). However, the clinical management of KOA combined with patellar dislocation presents greater challenges.

Patellar dislocation refers to the displacement of the patella from the femoral trochlear groove during movement, accounting for 3% of all knee joint injuries and being more prevalent in females (13, 14). Based on disease status and the degree of patellar dislocation, it can be further classified into congenital patellar dislocation, acute patellar dislocation, recurrent patellar dislocation, fixed patellar dislocation, HPD, etc (15–17). HPD is characterized by patellar dislocation during knee flexion and spontaneous reduction upon full extension. The pathogenesis of HPD primarily includes quadriceps contracture, lateral structural fibrosis and contracture, femoral trochlear dysplasia, lateral deviation of the tibial tubercle, genu valgum, and patella baja (18–20). Currently, clinical treatment approaches for HPD remain inconsistent and challenging. Treatment methods for adults include extensive lateral soft tissue release, proximal tibial tubercle transfer, medial tibial tubercle transfer, distal femoral osteotomy, trochleoplasty and MPFLr (21). Combined surgical approaches, such as double procedures [lateral retinacular release (LRR) combined with MPFLr], triple procedures (LRR, tibial tubercle transfer combined with MPFLr), and quadruple procedures (LRR, proximal tibial tubercle transfer, tibial tubercle medial transfer combined with MPFLr), have all demonstrated favorable outcomes in treating HPD (22–24). However, proximal tibial tubercle transfer may lead to complications such as patella alta (24). A systematic review reported an overall complication rate of 4.6% following tibial tubercle osteotomy, including nonunion risk at the osteotomy site (0.8%), tibial fracture risk (1.0%), wound-related complications (hematoma and dehiscence, 0.8%), and infection risk (1.0%) (25). There are relatively few reports on femoral derotational osteotomy for recurrent patellar dislocation. Blanke et al. employed MPFLr to treat recurrent patellar dislocation in patients with both increased femoral anteversion and normal femoral anteversion, finding no significant difference in outcome scores between the two groups (26). Sappey-Marinier et al. reported on 211 cases of recurrent patellar dislocation, demonstrating satisfactory clinical outcomes after MPFLr regardless of the presence of bony abnormalities such as increased femoral anteversion (27). The above research findings indicate that even in cases of recurrent patellar dislocation with increased femoral anteversion, isolated MPFLr can achieve satisfactory therapeutic outcomes. Currently, trochleoplasty mainly includes three surgical techniques: trochlear wedge resection, trochlear rectangular resection, and resectional trochleoplasty. However, these three techniques have several limitations, including high surgical complexity, prolonged operation durations, significant trauma, disruption of the original bony structure of the femoral trochlea, damage to the trochlear cartilage, and extended recovery periods, as well as complications such as nonunion or delayed union of osteotomy and post-traumatic arthritis (28, 29). This demonstrates that MPFLr holds considerable advantages in treating patellar dislocation. Current biomechanical studies have shown that the MPFL serves as the primary restraining factor against patellar dislocation (30, 31). MPFLr has now become the main surgical approach for managing chronic patellar instability (32). Although a systematic review (33) indicated that isolated MPFLr does not show clinical differences compared to LRR combined with MPFLr, the latter demonstrates higher Lysholm, Kujala, and Tegner scores. Moreover, a tight MPFL graft increases patellofemoral joint contact pressure, while LRR effectively reduces excessive patellofemoral joint pressure caused by MPFLr (34). Additionally, we believe that the patient's prolonged prolonged of HBD has led to fibrosis of the surrounding lateral patellar tissues and muscle tension.Therefore, after releasing the lateral retinaculum of the patella, the patella can be freed from the surrounding fibrotic tissues and muscles. Among the medial soft tissues that prevent lateral patellar dislocation, the MPFL contributes 50%–60% (35). Consequently, performing MPFLr following LRR can more effectively alleviate symptoms of patellar lateral dislocation.

The combination of KOA with patellar dislocation is relatively rare in clinical practice. There is currently no standardized treatment protocol for KOA combined with different types of patellar dislocation, with the majority of reported cases involving KOA with fixed patellar dislocation. Niu Ming et al. reported 12 cases of severe KOA with fixed patellar dislocation treated with TKA combined with overlapping suture of the medial parapatellar soft tissue structures, demonstrating satisfactory short- to mid-term clinical outcomes (36). Similarly, Gu Xinfeng et al. reported on 6 cases of KOA with fixed patellar dislocation treated using TKA in conjunction with extensive lateral structure release and medial retinaculum tightening. This approach resulted in significant improvement in the femorotibial angle and pain relief, with no extension lag observed at the 1-year follow-up (37). Xie Hongbin et al. reported a case of TKA combined with tibial tubercle osteotomy for KOA with fixed patellar dislocation, successfully reconstructing the knee extensor mechanism and achieving notable improvement in passive joint mobility and valgus deformity postoperatively (9). David et al. reported a case of TKA combined with vastus medialis muscle transposition and tibial tubercle transfer for KOA with fixed patellar dislocation, with satisfactory mid-term outcomes. The above-mentioned surgical approaches represent the current common clinical methods for treating KOA with fixed patellar dislocation, but only short- to mid-term clinical efficacy has been reported thus far, while long-term outcomes require further investigation. For patients with prolonged patellar dislocation who exhibit no relevant clinical symptoms, some literature suggests that patellar reduction may be unnecessary, as the knee joint function has already adapted well to the dislocated state (4, 38, 39). However, the patella serves as a crucial component of the knee extensor mechanism and acts as the fulcrum for the quadriceps lever. If the patella remains unreduced during TKA, active knee extension becomes impossible, making it difficult to maintain the stability of the knee prosthesis and resulting in limited range of motion. Therefore, most scholars advocate for patellar reduction (3, 37, 40). Takehiko et al. reported a case of valgus knee with osteoarthritis and patellar dislocation, where TKA combined with MPFLr achieved excellent clinical outcomes (41). We similarly believe that this approach presents an excellent opportunity for patellofemoral joint reduction and reconstruction of the extensor mechanism. Regardless of the preoperative functional adaptation of the patient's knee joint, patellar reduction and reconstruction of the extensor mechanism should be performed.

In this case, the preoperative radiograph indicated the presence of left femoral neck shortening and external rotation of the femur, suggesting that the lateral side of the trochlea is positioned more posteriorly. This may be considered to result from malalignment of the femoral torsion following intramedullary nailing, which constitutes a significant risk factor for patellar dislocation. Furthermore, the absence of the MPFL due to trauma further contributed to the occurrence of patellar dislocation. Considering the absence of the MPFL in the patient and the presence of adipose tissue, intraoperative findings indicated persistent patellar dislocation upon knee flexion, even after releasing the lateral patellar retinaculum and the lateral femoral tendon. Consequently, we chose to perform a simultaneous TKA combined with MPFLr to restore the knee extensor mechanism. Postoperatively, no patellar dislocation was observed. Considering the patient's advanced age, poor bone quality, weak quadriceps and hamstring muscles, anti-osteoporosis treatment was administered post-surgery, along with early guided rehabilitation exercises for periarticular muscle strengthening. At the seven-month follow-up, the patient reported satisfaction with the surgical outcome, demonstrating a normal gait, a stable and pain-free knee joint, no patellar dislocation during knee flexion, and radiographic evidence of well-positioned prostheses. Comparing the patient's preoperative and postoperative radiographs, it can be observed that the preoperative 10°varus alignment was reduced after the cutting at 6°during TKA. The femoral prosthesis was positioned close to the patient's native rotation. Correcting varus by rotating the prosthesis generates lateral shift of the lateral trochlear region, enabling the lateral trochlear wall to provide slightly more support to the patella during flexion. Although this effect is subtle and based only on visual assessment from radiographs, it could potentially contribute to maintaining patellar alignment and thereby slightly reduce the load on the MPFLr.

## Conclusion

In summary, the application of TKA combined with MPFLr for treating KOA complicated by HPD successfully reconstruct this patient's knee extensor mechanism, alleviate pain, improve knee joint mobility, and significantly enhance her quality of life. TKA combined with MPFLr may be suitable for patients with KOA and HPD, addressing both severe KOA and pre-existing patellar dislocation or malalignment. This may represents a clinically effective surgical approach. However, our report does have certain limitations. Due to the rarity of such cases, we only performed this treatment on a single patient. Although the clinical outcomes were remarkable, it remains necessary to increase the number of cases and extend follow-up periods to comprehensively evaluate the efficacy of this treatment approach and identify any potential complications.

## Data Availability

The original contributions presented in the study are included in the article/Supplementary Material, further inquiries can be directed to the corresponding authors.
